# Neutralizing and IgG Antibodies against Simian Virus 40 in Healthy Pregnant Women in Italy

**DOI:** 10.1371/journal.pone.0110700

**Published:** 2014-10-21

**Authors:** Manola Comar, Connie Wong, Mauro Tognon, Janet S. Butel

**Affiliations:** 1 Medical Science Department, University of Trieste, Trieste, Italy; 2 Institute for Maternal and Child Health – IRCCS “Burlo Garofolo”, Trieste, Italy; 3 Department of Molecular Virology and Microbiology, Baylor College of Medicine, Houston, Texas, United States of America; 4 Department of Morphology, Surgery and Experimental Medicine, Section of Pathology, Oncology and Experimental Biology, School of Medicine, University of Ferrara, Ferrara, Italy; University Hospital San Giovanni Battista di Torino, Italy

## Abstract

**Objective:**

Polyomavirus simian virus 40 (SV40) sequences have been detected in various human specimens and SV40 antibodies have been found in human sera from both healthy individuals and cancer patients. This study analyzed serum samples from healthy pregnant women as well as cord blood samples to determine the prevalence of SV40 antibodies in pregnancy.

**Methods:**

Serum samples were collected at the time of delivery from two groups of pregnant women as well as cord bloods from one group. The women were born between 1967 and 1993. Samples were assayed by two different serological methods, one group by neutralization of viral infectivity and the other by indirect ELISA employing specific SV40 mimotopes as antigens. Viral DNA assays by real-time polymerase chain reaction were carried out on blood samples.

**Results:**

Neutralization and ELISA tests indicated that the pregnant women were SV40 antibody-positive with overall prevalences of 10.6% (13/123) and 12.7% (14/110), respectively. SV40 neutralizing antibodies were detected in a low number of cord blood samples. Antibody titers were generally low. No viral DNA was detected in either maternal or cord bloods.

**Conclusions:**

SV40-specific serum antibodies were detected in pregnant women at the time of delivery and in cord bloods. There was no evidence of transplacental transmission of SV40. These data indicate that SV40 is circulating at a low prevalence in the northern Italian population long after the use of contaminated vaccines.

## Introduction

Simian virus 40 (SV40), a monkey polyomavirus, was discovered in 1960 as a contaminant of poliovirus vaccines which had been produced in naturally infected macaque kidney cells [1]. SV40-contaminated vaccines were inadvertently administered to millions of recipients between 1955 and 1963, providing a documented source of human exposure to SV40 [2]–[4]. The virus is widely recognized as having potent cell transforming ability *in vitro* and oncogenic activity in experimental animals [4]–[7]. This activity raised a concern that SV40 might cause human infections and perhaps contribute to cancer development.

SV40 DNA sequences have been detected in blood, urine, and tissue samples from healthy individuals [8]–[23]. SV40 DNA also has been found in association with several types of human cancer, including brain tumors, mesotheliomas, osteosarcomas, and lymphomas [8], [10], [11], [13], [24]–[39]. However, the International Agency for Research on Cancer decided recently that there was not enough firm evidence to classify SV40 as a carcinogenic viral agent of humans [40]. Although there is strong evidence that infections have occurred in certain populations in different geographic regions, more studies are needed of the current prevalence of SV40 infections in humans and the natural history of those infections [3], [4], [39], [41], [42].

Seroprevalence surveys are a common approach to examining the distribution of a virus within a host population. Neutralization assays, the most highly specific method for detection of viral antibodies in human sera, have been used in many studies [4], [9], [43]–[48]. SV40 seroprevalences generally have been in the range of 5–8%, although higher rates were detected in children who had received kidney transplants, in a group of HIV-positive men, and in Hispanic women in Texas (USA) [9], [44], [48]. However, this methodology is time consuming, labor intensive, lengthy, expensive, and requires specialized skills. Because of these limitations, neutralization assays are not practical for large epidemiological studies. Detection of SV40 antibodies has been attempted using enzyme immunoassays and SV40 virus-like particles or soluble capsid proteins. In those assays, all binding antibodies are measured, including non-neutralizing ones and those that recognize cross-reacting antigens on BK virus (BKV) and JC virus (JCV), resulting in some non-specificity concerns [49]–[51]. A newly developed ELISA employing specific SV40 synthetic peptides mimicking epitopes of viral capsid proteins VP1–3 seems to circumvent those problems [52]–[57]. Recent studies with this new assay have documented SV40 antibodies in Italian populations with estimated seroprevalences of 10–18%. Higher prevalences have been observed among patients with mesotheliomas and glioblastomas [53], [57].

Both DNA-based studies and serological surveys have detected SV40 markers in individuals too young to have been exposed to SV40-contaminated vaccines. These observations suggest that SV40 is being horizontally transmitted in humans. Maternal-infant transmission has been hypothesized to be a possible route of transmission of polyomaviruses [58]. This has been shown to occur in animal models, but no data substantiate that mode of transmission in humans [59]–[64]. This study was designed to determine the prevalence of SV40 antibodies in pregnant women and in their newborns and to examine the possibility of transplacental transmission of the virus.

## Materials and Methods

### Samples

Serum samples were collected at the time of delivery from a total of 233 healthy pregnant women and 100 matched cord blood samples from newborns. The enrolled women and their offspring were all of Caucasian origin and all the women were in their first pregnancy. Maternal serum collections represented two cohorts (Cohort A, n = 123, and Cohort B, n = 110), from Trieste and Ferrara, Italy, respectively. Cord blood samples [Cohort A(cb), n = 100] were collected at time of delivery of mothers in Cohort A. The demographics of study participants are shown in Table 1. The first group of maternal serum samples (Cohort A, n = 123) and a portion of the cord blood samples (n = 13) were analyzed for SV40 neutralizing antibodies in the Department of Molecular Virology and Microbiology, Baylor College of Medicine, Houston, Texas, USA, using a plaque-reduction neutralization assay [44], [47], [48]. The remaining cord blood samples (n = 87) were assayed at the University of Trieste, Trieste, Italy, using a neutralization test of inhibition of viral cytopathic effects (CPE) [52]. The other set of serum samples from pregnant women (Cohort B, n = 110) was tested by an indirect ELISA using specific mimotopes from SV40 viral capsid proteins at the University of Ferrara, Ferrara, Italy [52]. At the time of enrollment, study participants provided written informed consent. The study was approved by the Institutional Review Board for Human Subject Research at Baylor College of Medicine, the Institutional Scientific Board of the Burlo-Garofolo Children’s Hospital, Trieste, and the County Ethical Committee, Ferrara.

**Table 1:** Demographic characteristics of pregnant women in Italy analyzed for SV40 seropositivity.

Characteristics	Cohort A	Cohort B
Total subjects	123	110
Dates enrolled	2004–2005	2006–2011
Age, mean (range), years	30 (25–37)	36 (30–42)
Race, Caucasian	123	110
First pregnancy	123	110
Cord blood from newborns	100	0
Geographic location	Trieste, Italy	Ferrara, Italy

### SV40 neutralization assays

Two neutralization assays were used to detect SV40 serological reactivity. A plaque-reduction assay was performed using TC-7 cells as previously described [44], [47], [48]. Briefly, heat-inactivated serum samples were diluted in Tris-buffered saline (TBS; pH 7.4) and mixed with equal volumes of SV40 diluted to contain approximately 100 plaque-forming units (PFU) per 0.1 ml. Controls for each assay included: virus only, normal serum control (virus mixed with serum lacking SV40 antibodies), positive serum control (virus mixed with rabbit hyperimmune serum having high neutralizing activity), and uninoculated cells. Samples were incubated at 37°C for 30 min. TC-7 cell monolayers in 60-mm^2^ tissue culture plates were then inoculated with 0.2 ml of virus–serum mixture per culture and inocula were adsorbed for 2 h. Cell monolayers were overlaid with a mixture of agar and enriched Eagle medium. Plaques were counted on day 15. Each sample was tested in triplicate. Initial tests were carried out using final serum dilutions of 1∶10, and positive samples (those that reduced the number of plaques by ≥50% compared to the virus-only control) were titered in repeat experiments to determine the endpoint neutralization titers. A modified neutralization assay using CV-1 cells and based on inhibition of viral CPE was previously described [52]. Sera diluted 1∶20 were mixed with 5 × 10^4^ PFU of SV40. Following a 30-min incubation, the mixture was applied to CV-1 monolayers for 2 h. The cell monolayers were then washed three times, fresh media was added, and cultures were observed for CPE for 21 days. Samples were tested in duplicate and neutralizing activity was evidenced by inhibition of CPE.

### Indirect enzyme-linked immunosorbent assay (ELISA) with SV40 synthetic peptides

An indirect ELISA developed to detect SV40-specific antibodies using two synthetic peptides based on sequences of SV40 viral proteins VP1 and VP2/3 was performed as detailed previously [52], [54]. These peptides were VP1 B: NH2- NPDEHQKGLSKSLAAEKQFTDDSP- COOH; and VP2/3 C: NH2- IQNDIPRLTSQELERRTQRYLRD- COOH. The human peptide hNPS, sequence SFRNGVGTGMKKTSFQRAKS, was employed as a negative control peptide [52], [56]. The synthetic peptides were synthesized by standard procedures and were purchased from UFPeptides s.r.l., Ferrara, Italy. Comparative computer-assisted analyses using a BLAST program were carried out previously with the SV40 VP peptides B and C and the corresponding amino acid sequences of the new human polyomaviruses (HPyV) and hundreds of different BKV and JCV serotypes [52], [54]. Results indicated a low homology for the BKV and JCV prototypes and other polyomaviruses. This assay does not cross-react with BKV and JCV hyperimmune sera (negative controls) [52], [54]. Hyperimmune sera against SV40 and BKV were obtained in rabbits that had been inoculated with purified viral stocks as reported previously, whereas SV40-positive human sera were from a serum collection [52]. The serum against JCV was kindly provided by Dr. E. Major, NIH, Bethesda, MD, USA [52], [54]. Briefly, each well was coated with 5 µg of peptide in 100 µl of Coating Buffer (Candor Bioscience, Germany) at 4°C for 16 h, followed by 200 µl/well of Blocking Solution (Candor Bioscience) at 37°C for 90 min. Wells were covered with 100 µl of test serum diluted 1∶20 in Low Cross-Buffer (Candor Bioscience). The secondary antibody was a goat anti-human or anti-rabbit IgG heavy and light chain-specific peroxidase conjugate (Calbiochem-Merck, Germany) diluted 1∶10,000 in Low Cross-Buffer. Samples were treated with 2,2-azino-bis 3-ethylbenzthiazoline-6-sulfonic acid (ABTS) solution (Sigma-Aldrich, Milan), for 45 min at room temperature and then read spectrophotometrically at a wavelength of 405 nm. The cut-off was determined in each assay by an OD reading of two negative controls, added to the standard deviation and multiplied three times (+3SD). Positive and negative controls included immune rabbit serum containing SV40 antibodies, immune sera containing BKV or JCV antibodies, and three human serum samples known to be SV40 negative. Each sample was analyzed in duplicate three times. Sera were considered to be SV40 antibody-positive if they reacted with both peptides three different times by indirect ELISA. SV40 antibody titers were determined by testing serial dilutions of positive sera.

### Viral DNA assay

DNA was extracted from 500 µl of total cord blood and maternal peripheral blood samples using a commercial kit (QIAamp Tissue Kit; Qiagen, Mannheim, Germany) following the manufacturer’s instructions. DNA was eluted in 100 µl TE buffer and stored at –80°C until analyzed. Real-time polymerase chain reaction (PCR) assays for SV40 and BKV DNA were carried out using primers and probes and assay conditions as described [23], [65]. In brief, real time PCR was performed using the ABI PRISM 7000 Sequence Detection System according to the manufacturer’s recommendations. Primers and probes were designed to detect sequences in the conserved N-terminal region of the large T-ag gene. Plasmids containing full-length viral genomes, SV40-B2E and BKV-Dunlop-1, were used as positive controls while negative controls were reactions without DNA. For clinical samples, 10 µl of DNA per reaction were tested in duplicate. For standards, 10 µl of DNA which contained viral copy numbers ranging from 10^7^ copies to 10° copies were also tested in duplicate. Real time PCR was performed for the single copy human RNAse P gene to establish the suitability of DNA test samples for analysis [65].

### Statistical analysis

Statistical significance between two groups was determined using Student’s t test. A finding of *p*≤0.05 was considered to be statistically significant. The profile of serum antibody reactivity to SV40 mimotopes was statistically analyzed using Anova and Newman-Keuls Comparison tests [52], [54], [56], [57], [66].

## Results

### Serum neutralizing antibodies to SV40 in pregnant women and their newborns

Serum samples were collected from 123 pregnant women at the time of delivery (Cohort A) as well as cord blood samples from 100 of their newborns [Cohort A(cb)]. These samples were assayed for SV40-specific antibodies using neutralization assays (Table 2). Plaque-reduction assays revealed that 13/123 (10.6%) of the maternal sera were positive for SV40 neutralizing antibodies. In the majority of positive samples the neutralizing antibody titer was low (1∶10), but two samples had titers of 1∶40 and 1∶160, respectively.

**Table 2:** Detection of SV40 antibodies in sera from pregnant women and in cord blood.

Cohort	Type of sample	Assay	Numbertested	Number SV40antibody-positive (%)
A	Pregnant women sera	Neutralization plaque reduction	123	13 (10.6)
B	Pregnant women sera	Indirect ELISA	110^a^	14 (12.7)
A(cb)^b^	Cord blood	Neutralization plaque reduction, inhibition of CPE	100	7 (7.0)

a Some of these subjects (n = 94) were included in a previous report [53].

b Cord bloods were collected at time of delivery of women in Cohort A.

Among the newborn cord bloods, 7 of 100 (7.0%) contained detectable SV40 neutralizing antibodies at low titers (Table 2). The antibody-positive samples were in the subset analyzed by the plaque-reduction neutralization assay (Materials and Methods). No matches between positivity of maternal sera and cord bloods were observed.

### Absence of viral DNA

Viral DNA assays by real time PCR were carried out on DNAs recovered from maternalperipheral blood (Cohort A, n = 123) or from cord blood [Cohort A(cb), n = 100] samples. No SV40 or BKV DNA was detected in any of the samples.

### IgG antibodies reacting with SV40 mimotopes in serum samples from pregnant women

Serum samples from additional pregnant women (Cohort B, n = 110) were analyzed by indirect ELISA for the presence of IgG class antibodies reactive against SV40 VP mimotopes, VP1 B and VP2/3 C [52]. In this group of sera, the prevalence of specific SV40 antibodies was determined to be 12.7% (14/110) (Table 2). Samples seropositive for SV40 VP1 B were also found to be positive for SV40 VP2/3 C, and vice versa, with negligible exceptions. Sera were considered to be SV40-positive when reactive with both VP1 B and VP2/3 C peptides. The difference in prevalence of SV40 antibodies detected in Cohort A by neutralization (10.6%) and in Cohort B by ELISA (12.7%) was not statistically significant (p>0.05).

### Age distribution of SV40 antibodies

SV40 seropositivity rates were compared for both birth cohorts of pregnant women (Table 3). The groups were subdivided in several ways to approximately equalize subject numbers in Cohort A and in Cohort B and in the span of birth years for comparison purposes. SV40 antibody-positive samples were distributed among all birth cohorts. None of the differences in seropositivity for the various comparisons was statistically significant (*p*>0.05).

**Table 3:** Age distribution of SV40 antibodies in pregnant women.

Year of birth	Cohort A^a^		Cohort B^b^	
	No. SV40 antibody positivesera/No. sera tested	% SV40 positive	No. SV40 antibody positivesera/No. sera tested	% SV40 positive
1967–1970	7/29	24.1	4/36	11.1
1971–1974	2/25	8.0	4/38	10.5
1975–1978	1/15	6.7	4/20	20.0
1979–1980/1993^c^	3/54	5.6	2/16	12.5
Total:	13/123	10.6	14/110	12.7
Comparison of birth groups:				
1967–1972	8/43	18.6	6/60	10.0
1973–1980/1993	5/80	6.2	8/50	16.0
1967–1975	9/60	15.0	9/83	10.8
1976–1980/1993	4/63	6.3	4/27	14.8
1967–1978	10/69	14.5	12/94	12.8
1979–1980/1993	3/54	5.6	2/16	12.5

a Samples collected 2004–2005.

b Samples collected 2006–2011.

c Cohort A contained subjects born through 1980; cohort B contained subjects born through 1993.

## Discussion

This study provides SV40 seroprevalence data for women from a geographic region in northeastern Italy. Evidence of SV40 infection has been observed in children with immune defects and in adult patients with mesothelioma or colon cancer from this area [23], [25], [67], [68]. This study found that overall 10.6% of pregnant women were seropositive for SV40 neutralizing antibodies and 12.7% were positive for SV40-specific IgG antibodies (Tables 2, 3). The detection of SV40 antibodies in cord blood from 7.0% of newborns of mothers in Cohort A provides another indication of maternal seropositivity. These findings substantiate previous observations of SV40 antibodies in Italian blood donors and other populations [52]–[57].

The overall prevalence of SV40 IgG antibodies detected in healthy pregnant women by ELISA (12.7%) was similar to the estimate based on neutralization assays (10.3%); the difference was not statistically significant. Taken together, these data provide a comprehensive analysis of SV40 seroprevalence in pregnancy in northern Italy. Whereas it would have been desirable to perform both immunological tests on the same sera, sample availability precluded that approach.

The pregnant women in Cohort A of this study were born from 1967 to 1980 and those in Cohort B from 1967 to 1993. Italy started using the live, attenuated Sabin polio vaccine strains in 1964 [16], [69]. As contaminated poliovaccines were supposedly virus-free from SV40 after 1963, it is unlikely that vaccine exposure explains that SV40 antibody positivity. An earlier study of Italian organ donors detected SV40 DNA in blood samples at similar prevalence in birth cohorts born from 1924 to 1983 [16]. These data together indicate that other sources of SV40 exposure must exist in the human population and that infections likely reflect human-to-human spread [3], [4], [16], [39]. Interestingly, fecal excretion of polyomaviruses BKV and SV40 has been detected in one-year-old children in Mexico City, suggesting that the gastrointestinal tract may be a site of polyomavirus persistence in humans and that fecal contamination could be a source of virus transmission [3], [70].

SV40 antibody titers detected in maternal sera tended to be low, with titers that were comparable to those reported in other studies of SV40 neutralizing antibody responses in humans [9], [44]–[48], [71]–[74]. The titers could be a reflection of a weak human immune response to SV40, of limited replication of the virus in human cells, and/or of waning of SV40 antibodies over time [75]. The presence of SV40 antibodies in some cord bloods is not unexpected as it is known that maternal IgG antibodies can pass through the placenta to a fetus during pregnancy. The discordance observed in antibody detection in maternal sera and matched cord bloods probably reflects the low antibody titers and the complexities of the neutralization assays.

The transient immunosuppression associated with pregnancy could allow reactivation of latent viruses, including polyomaviruses, raising the theoretical possibility of transplacental transmission from mother to fetus. More frequent detection in urine samples from pregnant women has been reported for BKV but not for other polyomaviruses [76], [77]. Transplacental transmission of polyomaviruses can occur in animals, including murine polyomavirus in mice and rats and SV40 in hamsters and rhesus monkeys [59]–[64]. Although BKV DNA has been detected in autopsy tissues of aborted human fetuses, evidence of transplacental transmission is seldom found; if such transmission occurs, it must be a rare event [77]–[84]. In this study there was no evidence of transplacental transmission as no SV40 viral DNA was detected in maternal peripheral blood or in cord blood. It is possible that transmission *in utero* might occur if the mother suffered a primary viral infection during pregnancy, as higher levels of replicating virus would be present at that time.

In summary, the detection of SV40 antibodies in pregnant women in northern Italy strengthens the evidence that SV40, or a closely related unknown virus, is causing infections in humans years after the use of SV40-contaminated vaccines. The significance of those infections to the health of the affected individuals is unknown.
